# Radiation Exposure and Lifetime Attributable Risk of Cancer Incidence and Mortality from Low- and Standard-Dose CT Chest: Implications for COVID-19 Pneumonia Subjects

**DOI:** 10.3390/diagnostics12123043

**Published:** 2022-12-05

**Authors:** Mandeep Garg, Vahid Karami, Javad Moazen, Thomas Kwee, Ashu Seith Bhalla, Daryoush Shahbazi-Gahrouei, Yu-Hsuan Joni Shao

**Affiliations:** 1 Department of Radiodiagnosis & Imaging, Post Graduate Institute of Medical Education and Research, Chandigarh 160012, India; 2 Clinical Research Development Unite, Ganjavian Hospital, Dezful University of Medical Sciences, Dezful 6461653476, Iran; 3 Infectious and Tropical Diseases Research Center, School of Medicine, Dezful University of Medical Sciences, Dezful 6461653480, Iran; 4 Department of Radiology, Nuclear Medicine and Molecular Imaging, University Medical Center Groningen, Hanzeplein, 9700 Groningen, The Netherlands; 5 Department of Radiodiagnosis, All India Institute of Medical Sciences, New Delhi 110029, India; 6 Department of Medical Physics, Faculty of Medicine, Isfahan University of Medical Sciences, Isfahan 8174673461, Iran; 7 Graduate Institute of Biomedical Informatics, College of Medical Science and Technology, Taipei Medical University, Taipei 106, Taiwan

**Keywords:** computed tomography, chest, radiation risk, COVID-19, cancer

## Abstract

Since the novel coronavirus disease 2019 (COVID-19) outbreak, there has been an unprecedented increase in the acquisition of chest computed tomography (CT) scans. Nearly 616 million people have been infected by COVID-19 worldwide to date, of whom many were subjected to CT scanning. CT exposes the patients to hazardous ionizing radiation, which can damage the genetic material in the cells, leading to stochastic health effects in the form of heritable genetic mutations and increased cancer risk. These probabilistic, long-term carcinogenic effects of radiation can be seen over a lifetime and may sometimes take several decades to manifest. This review briefly describes what is known about the health effects of radiation, the lowest dose for which there exists compelling evidence about increased radiation-induced cancer risk and the evidence regarding this risk at typical CT doses. The lifetime attributable risk (LAR) of cancer from low- and standard-dose chest CT scans performed in COVID-19 subjects is also discussed along with the projected number of future cancers that could be related to chest CT scans performed during the COVID-19 pandemic. The LAR of cancer Incidence from chest CT has also been compared with those from other radiation sources, daily life risks and lifetime baseline risk.

## 1. Introduction

Computed tomography (CT) has come a long way since its introduction in 1972 and it has revolutionized diagnostic radiology [1]. CT is a noninvasive imaging modality that creates cross-sectional and three-dimensional (3D) images of the internal anatomical structures of the body, leading to improved diagnosis, and in turn, saving many lives [2,3]. There has been an exponential increase in the number of CT examinations in the last two decades. In 2019, more than 90 million CT scans were performed in the United States [4], up from 85 million in 2011 [5], 62 million in 2007 [6] and 57 million in 2000 [7]. CT is the major source of radiation exposure to the general population from medical imaging, which is evident from the fact that while CT represents only ~6.3% of all diagnostic radiologic procedures, it contributes to ~43.2% of the collective radiation dose given to the patients [8]. This has become a matter of growing concern as these harmful ionizing radiations can lead to DNA damage, mutagenesis and carcinogenesis in the exposed individuals [9].

Some epidemiological studies have shown a small yet significant increase in cancer risk at typical CT doses [10,11,12,13,14,15]. One report estimated that 0.9% of cancer cases in the United States could be related to low-dose diagnostic X-rays performed between 1991–1996 [16]. Given the increasing use of CT, Brenner and Ha`ll translated these figures to 1.5–2% of the 2007 United States cancer cases [6]. Since the novel coronavirus disease 2019 (COVID-19) pandemic, the role of chest CT has garnered increased attention for screening, diagnosis and management of patients with suspected or known COVID-19, as well as for monitoring the disease progress and its complications [17,18]. To date, more than 616 million cases of COVID-19 have been identified worldwide [19,20], many of whom were subjected to CT scanning [21] and some even underwent repeat CT examinations ranging between 2–8 scans [21,22,23,24,25]. The dramatic increase in the number of CT scans in a short span of time has raised concerns about patient safety [21].

The awareness and understanding of radiation dose levels and risks associated with medical imaging tests are still limited [26]. The current review article briefly describes what is known about the health effects of radiation, the lowest dose for which there exists compelling evidence about increased radiation-induced cancer risk and the evidence regarding this risk at typical CT doses. We also describe the lifetime attributable risk (LAR) of cancer from low- and standard-dose chest CT performed in COVID-19 subjects and the projected number of future cancers that could be related to chest CT scans performed during the COVID-19 pandemic. The LAR of cancer incidence from chest CT has also been compared with those from other radiation sources, daily life risks and lifetime baseline risk. We conducted an extensive literature review by searching various online databases: Medline, PubMed, Web of Science, Scopus, ResearchGate, medRxiv, bioRxiv and Google scholar search engine.

## 2. Health Effects of Radiation Exposure

The health effects of ionizing radiation can be divided into stochastics and deterministic effects. Stochastic effects suggest that exposure to radiation, even at low doses, may cause damage to the genetic material in cells that can result in cancer induction or hereditary disease in the future [27]. These are not seen immediately, but over a lifetime, and sometimes manifest several decades after the exposure. Stochastic effects are unpredictable, random events in nature with no specific threshold [28]. The probability of stochastic effects, rather than its severity, is assumed to increase linearly with the increasing dose [29,30]. Prevention of stochastic effects is not possible in practice, though dose limits are established to reduce their chance of occurrence [27].

Deterministic effects, on the other hand, are seen when patients are exposed to high doses of radiation over a short span of time [28]. These have a threshold dose, below which they do not occur; however, once the threshold is exceeded, the severity of the outcome increases [29]. Skin erythema, cataract, hair loss and burns are examples of such effects [8,28,29]. However, these effects are seldom seen with low-dose diagnostic imaging modalities such as CT, except for a few sporadic incidences of gross medical error [31].

The general population is at some risk for cancer and associated mortality during their lifetime, even without being exposed to medical radiation. This risk is called the lifetime baseline risk (LBR) for cancer. In the United States, the sex-averaged LBR of cancer incidence and mortality (including solid cancers and leukemia) is about 42% and 20%, respectively [8]. According to the American Cancer Society, based on 2016–2018 data, the average lifetime risk of developing cancer from other causes stands at 40.14 and 38.7% in men and women, respectively [32]. The additional cancer risk above and beyond LBR due to radiation exposure is called the lifetime attributable risk (LAR) [33,34]. Table 1 and Table 2 represent qualitative approaches to communicate the LAR of cancer incidence and mortality compared to LBR [8]. 

The LAR is calculated using risk estimation models derived from epidemiological studies, mainly Japanese atomic bomb survivors, taking into account a conservative assumption that there is a ‘linear-no-threshold’ (LNT) relationship between radiation exposure and cancer risk at all dose levels, even near zero [8,28,29,35]. The foundation of the LNT model of dose–response is based on statistical extrapolation of the risks at high-dose (where the risks are observable with epidemiological evidence) to low-dose radiation (where the risks are not observable) [33,36]. The LNT postulates that (i) a single ionization at any dose, however small it may be, has the potential to initiate complex processes that can cause stochastic health effect; (ii) the effects increase linearly with the increase in radiation dose; and (iii) these effects are cumulative over lifetime, and the sum of several small exposures carries the same potential to produce these effects as a single large exposure of equal dose value [37].

However, various authors and professional organizations, including the Health Physics Society [38], United Nations Scientific Committee on the Effects of Atomic Radiation [39], United States Nuclear Regulatory Commission [40] and American Nuclear Society [41], have challenged and debunked LNT theory, considering it only a mathematical formula that calculates the theoretical and hypothetical risk.

Many other studies have also deprecated the fundamental assumption and historical foundation of the LNT model, especially for low-dose radiation, as LNT theory ignores the body’s natural ability to repair damaged DNA and elimination of aberrant cells [42,43]. Moreover, it has also been contested that most of the studies supporting the LNT theory lack merit, as they are not evidence-based and ignore radiobiology [44].

The existence of three other dose–response models (hypersensitivity, threshold and hormetic) for estimating the carcinogenic risks of radiation makes things even more complicated. The hypersensitivity model suggests a greater risk than those from the LNT model at low-dose radiation [45]. The ‘threshold’ model assumes that there exists a latency threshold below which small exposures of radiation are harmless [44], and the ‘hormetic’ model suggests that low-dose radiation, on the contrary, may help to prevent rather than cause cancer, by stimulating the body’s natural anticancer mechanisms that are otherwise not activated in the absence of radiation [43,46]. Stimulation of such adaptive processes not only helps in the repair/elimination of the cells affected by radiogenic damage, but also of the pre-existing (pre-exposure), steady-state damaged cells that are there in the body due to spontaneous biological damage. It is understandable, though, that such repair and/or removal may not be 100% efficient, but it is incorrect to completely omit these mechanisms from consideration.

**Figure 1:**
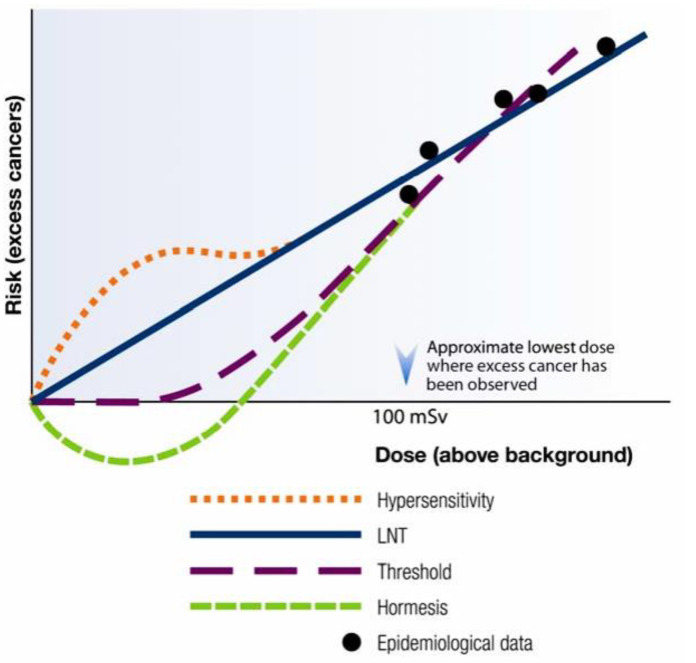
Different radiation risk models illustrating the estimated health risk at low levels of ionizing radiation (Reprinted with permission from Ref. [45]. 2022, Canadian Nuclear Safety Commission).

The various radiation dose–response models used to estimate the risk of cancer at low-dose (<100 mSv) radiation exposure are illustrated in Figure 1.

However, the National Council of Radiation Protection and Measurements (NCRP), based on a critical review of the recent epidemiological studies assessing dose–response at low-dose and low-dose rate radiation, recognized that the risks are small and uncertain. Nevertheless, it broadly supports the LNT theory for radiation protection purposes, as no better alternative dose–response model is available as of today [47]. Other regulatory bodies, such as the International Commission on Radiological Protection (ICRP) [27], the United States Environmental Protection Agency (EPA) [48], the United States Nuclear Regulatory Commission (NRC) [36] and the United States National Research Council (NRC) [33] also currently support LNT theory at low-dose radiation.

Another recent review of different dose–response models suggests that scientific evidence supports different biological mechanisms at low-dose radiation; however, they are still not fully understood. Moreover, even if there is an increased risk at low-dose radiation, it must be small, as there are no sufficient epidemiological data for an observable effect [49].

The relatively high magnitude of LBR of cancer incidence (~42%) in the general population makes it difficult to perform an epidemiological study with a large sample size to evaluate the risk of low-dose radiation with sufficient statistical power [50]. The sample size is proportional to the inverse square of the dose; thus, to quantify the risk of low-dose radiations with precision, larger epidemiological studies are required [51,52]. For example, if a sample size of 500 individuals is needed to quantify the risk of a 1000 mSv dose, to maintain the same statistical power and precision, a sample size of ~5 million subjects would be required for a 10 mSv dose [51]. Additionally, there are many uncertainties in estimating radiation risks due to several other factors, such as statistical uncertainty, application of risk estimation results in the population exposed to other radiation sources, the random nature of processes that cause cancer, insufficient data, a lack of idealized models to describe the nature of risks in exposed and non-exposed populations, and exposure to other cancer risk factors such as smoking [27,53]. The Biologic Effects of Ionizing Radiation (BEIR) VII report presented its best estimates for cancer incidence and mortality at low-dose radiation in human subjects (Table 3) [33]. These estimates are accompanied by 95% subjective confidence intervals that reflect the important sources of uncertainty, nearly by a factor of two.

With the given controversies and uncertainties in dose–response models, there is currently no consensus on LAR estimates for low-dose radiation exposures [8] and radiation protection policies [10]. It is likely that the risk of some cancers could be overestimated, while those of others is underestimated [51]. Moreover, a subset of individuals can be more susceptible and genetically predisposed to the carcinogenic effects of radiation, such as those with congenital/acquired genetic mutations or defective genes [54]. 

Thus, with the understanding of radiation-related cancer risk still evolving, and until the time we obtain clear answers, a conservative policy needs to be adopted to ensure patients’ safety by following the basic ALARA (as low as reasonably achievable) principle of radiation exposure through the process of justification and optimization [8].

## 3. Radiation Dose Quantities in CT

Several terms define the radiation dose related to CT scanning. The absorbed dose describes the amount of absorbed energy from ionizing radiations per unit mass. It is measured in gray, and one gray equals the absorption of one joule of radiation energy per kilogram of matter (J/Kg) [3].

The computed tomography dose index (CTDI) and dose-length product (DLP) are two commonly used descriptors to quantify the absorbed dose in a specific CT protocol. CTDI, measured in milligray (mGy), is a standardized measure of radiation dose output from a single gantry rotation [55,56]. It is measured by a 100 mm length pencil ionization chamber located at the center and several peripheral points of a 16 cm or 32 cm cylindrical Perspex phantom [56,57]. Since there is spatial variation across the scan plane in terms of dose, adjustments are needed by summing the 1/3 CTDI at the center and 2/3 CTDI at the periphery points to give the weighted CTDI (CTDI_w_). Volume CTDI (CTDI_vol_), the ratio of CTDI_w_ to pitch, describes the average radiation output within the scanned volume and takes into account the gaps or overlaps between consecutive X-ray beam rotations in helical scans [55,57]. However, it is limited to the comparison of doses delivered to individual patients, because it does not include the length of the scan. DLP, measured in mGy.cm, represents the overall radiation dose output delivered by a given scan protocol, and is calculated by multiplying the CTDI_vol_ by the total scan length [57,58]. It is important to realize that these dose descriptors are not a real measurement of patient dose, but that they are estimated indices for comparison of CT scanner radiation output and absorbed dose in standardized phantoms between different scan protocols and scanners [55].

Effective dose (ED), measured in mSv, is the sum of the equivalent doses to organs and tissues irradiated, each multiplied by a specific tissue weighting factor [27,35]. In CT, the effective dose is generally derived from DLP using appropriate sex- and age-specific conversion factors [59,60].

## 4. Cancer Risk at Low-Dose Radiation in Human Subjects

The evidence for radiation-induced cancer mainly comes from four groups: (a) Japanese atomic bomb survivors, (b) medically and (c) occupationally exposed individuals, and (d) individuals living in areas with high background radiation [61].

The life span study (LSS) among the cohort of atomic bomb survivors supports statistically high solid cancer incidence [10,62] and mortality [63] at 5–125 mSv of acute doses. For protracted exposures, some epidemiological studies support statistically significant increases in solid cancer incidence and mortality [64,65], including breast cancer [66], thyroid cancer [67] and leukemia [68], at <100 mSv doses. There is some human-based evidence supporting radiation-induced cancer at 10–50 mSv for acute exposures; and 50–100 mSv for protracted exposures [51]. However, it is important to understand that the risk of acute, instantaneous whole-body exposures (e.g., from atomic bombs) should be distinguished from the specific body part single-time/protracted exposure (from medical diagnostic imaging). Furthermore, it is noteworthy that for the same total dose, the estimated risk from protracted exposures is lower than those from acute exposures [69].

Since the 2006 BEIR VII report review of experimental and epidemiological data for cancer risk from low-dose radiation, some subsequent epidemiological studies have linked radiation exposure from CT with the risk of cancer increase [11,12,15,70], whereas other reports contended increased cancer risk [71,72,73]. A recent systematic review and meta-analysis comprising 26 epidemiological studies published from 2006–2017 with a total of 3.6 million individuals found excess cancer risk from ≤100 mGy doses [74]. Another more recent systematic review and meta-analysis comprising 24 epidemiological studies published from 2000–2019 including patients < 22 years old also reported a statistically significant excess cancer risk from CT scan [75]. Similarly, Pears et al. conducted a large-scale retrospective cohort study comprising a total of 178,604 children and young adults who underwent CT scanning in National Health Service (NHS) centers in Great Britain (1985–2002). None of these patients had a cancer diagnosis before the scan, and this study again found a positive correlation between radiation exposure from CT and the development of leukemia and brain tumors. Pediatric cases whose active bone marrow received a dose of ≥30 mGy in CT procedures were 3.2% more susceptible to develop leukemia, and those whose brain received a dose of ≥50 mGy were 2.8% more likely to develop brain tumors [70]. A more recent large population-based cohort study including 12,068,821 youths aged 0–19 years in South Korea also clearly supports increased cancer incidence from diagnostic low-dose radiation [76]. One report estimated that ~70 million CT scans performed in the United States in 2007 could translate into 29,000 future cancers and ~14,500 cancer deaths [77]. A similar report estimated that 4 million pediatric CT scans performed each year in the United States are related to 4870 future cancers [5]. Based on data from the United Kingdom and 14 other developed countries, Berrington de Gonzalez and Darby estimated that 0.6–3.2% of the cumulative risk of cancer could be related to diagnostic radiologic procedures [16]. The results of these studies are consistent with the ICRP recommendations that state “the absorbed dose to tissue from CT can often approach or exceed the levels known to increase the probability of cancer” [78].

Contrary to this, in another systematic, methodological review of 62 epidemiological studies published from 1975–2017 examining cancer risk from low-dose radiation, only 27 studies support cancer induction by doses < 200 mSv, whereas 35 studies did not support cancer induction at this dose range. Quality assessment of the methodological strengths of these studies revealed 25 studies with high methodological quality, of whom only 4 studies support cancer induction by doses < 200 mSv. Based on these findings, the authors concluded that exposure to cumulative doses up to 100 mSv (~10 CT scan) and possibly 200 mSv (~20 CT scan) does not increase cancer risk [79]. The latest update of solid cancer incidence among the LSS cohort of atomic bomb survivors (1958–2009) using a revised dosimetry system (DS02R1) and adjustment for smoking showed that for males, there was no statistically significant increase in cancer risk at <75 mSv doses, whereas for females, there was no evidence of a threshold dose below which there was no dose–response [10].

Nevertheless, despite the conflicting reports regarding estimates of cancer risk at a radiation dose of <100 mSv, some authors called for action due to significant cumulative exposure from recurrent CT scans [80,81]. One report including data from ~4.8 million CT scans from 4 institutions covering 324 sites during the period of 1–5 years identified 33,407 (1.33%) patients with a cumulative effective dose (CED) of ≥100 mSv and with a maximum reported dose of 1185 mSv [80]. Another report including data from ~3.2 million patients who experienced medical imaging tests during the period of 1–5 years at different sites across 26 countries found that the frequency of patients with CED ≥100 mSv was higher than previously estimated. It was estimated that an additional 0.9 million patients worldwide are subjected to CED ≥100 mSv annually [81].

## 5. Low- and Standard-Dose Chest CT in COVID-19: Radiation Exposure

The rapid spread of the pandemic prompted several healthcare providers and sites to develop low-dose chest CT protocols for COVID-19 subjects. Some studies reported an 88–91% reduction in effective dose without compromising the diagnostic image information in low-dose compared to standard-dose chest CT protocols [24,82,83]. Table 4 summarizes the main scan settings, radiation dose quantities and sex-averaged LAR of cancer incidence and mortality for a wide age range used in the literature for low-dose chest CT in COVID-19 subjects. For comparison, similar information for standard-dose chest CT is presented in Table 5. The most common technical parameters manipulated in low-dose protocols were tube potential (for young and pediatric patients, in particular) and most importantly, tube current (mA).

Table 4 and Table 5 represent that there is a large variation in radiation dose levels reported in the literature for chest CT, especially for low-dose protocols. The CTDI_vol_, DLP and effective dose varied between studies ~2–4-fold in standard-dose protocols; and ~9-fold in low-dose protocols. Based on data from 782 adult chest CT scans from 54 healthcare sites in 28 countries, Homayounieh et al. reported ~25-fold (1.5–38 mGy) and DLP ~42-fold (53–2231 mGy.cm) variation in CTDI_vol_, depending on the vendor, the number of detector rows, year of CT installation and image reconstruction techniques used [25]. Similar variations appear to exist for other CT study types as well. Smith-Bindman et al. reviewed 1100 CT scans of the head-and-neck, chest and abdomen-pelvis across 4 healthcare sites and reported a mean of 13-fold variation between the highest and lowest effective doses for a given study type [50]. The mean effective doses also differed 2–3-fold across the 4 sites [50].

Recently, the American association of physicists in medicine (AAPM) recommended a CTDI_vol_ ≤ 3 mGy, DLP ≤ 75 mGy.cm and ED ≤ 1 mSv for non-contrast low-dose chest CT for an idealized standard-sized patient [96] that is comparable with those from low-dose chest CT protocols used in the literature for screening COVID-19 subjects (Table 4).

## 6. Low- and Standard-Dose Chest CT in COVID-19: Radiation Risk

Several studies have addressed the LAR of cancer incidence and mortality from low- and standard-dose chest CT scans [89,90,91,92,97,98]. From Table 4 and Table 5, it is evident that the effective dose resulting from a low-dose chest CT for COVID-19 is much lower than that from standard-dose chest CT (0.20–1.8 mSv Vs. 2.20–8.70 mSv). The radiation-related risk is also expected to decline with low-dose CT, but not to zero, according to some reports, especially for smokers and young women [99,100]. Brenner estimated that a single, low-dose lung CT for cancer screening would increase the LAR of lung cancer incidence by 1–6 per 10,000 people, depending on the patient’s age at exposure, sex and smoking status [99]. In a similar study, Berrington de Gonzalez et al. reported that for never-smokers, the LAR of lung cancer mortality from annual low-dose lung CT screening aged 40–42 years was 1–3 per 10,000 people, while for smokers, there was a 2-fold increase in the risk [100].

Chest CT exposes several radiosensitive tissues such as the breasts, lungs and thyroid gland to radiation [89,101], and is shown to increase the risk of cancer induction in these tissues. A meta-analysis including seven studies assessing breast cancer induction by low-dose radiation from mammography or chest X-rays in women with familial or genetic predisposition of breast cancer found a 1.3-fold (Odd ratio = 1.3, 95% CI: 0.9–1.8) increased risk of breast cancer in <33 mSv doses. The risk of radiation-induced breast cancer was significantly higher in ages < 20 years and in women who received ≥2 exposures [102]. A pooled analysis of seven cohort studies assessing thyroid cancer after exposure to external radiation supports thyroid cancer increase at 10–90 mSv doses [103]. Overall, it is estimated that a standard-dose chest CT may increase the risk of breast cancer by 20–287 [89,90,92,97,98] and lung cancer by 22–152 [90,91,97,98] per 100,000 people.

Although chest CT has not been used frequently in the pediatric age group in COVID-19, it is noteworthy that for most types of cancers, such as breast, thyroid, brain, skin and leukemia, children are more sensitive to radiation than adults by a factor of 2–3 [8,104]. A subset of the pediatric population with specific genetic disorders such as ataxia-telangiectasia, AT-like disorder, dyskeratosis congenita, Seckel syndrome, Ligase IV syndrome, Werner’s syndrome, Nijmegen breakage syndrome and Fanconi anemia should be considered as “hyper-radiosensitive” [105]. Such vulnerable and susceptible individuals need special attention and justification before taking them for any radiological investigation involving ionizing radiation.

From Table 4 and Table 5, in general, the LAR of cancer incidence and mortality related to a single low-dose chest CT is estimated at 2–8 and 1–5 per 100,000 people, respectively. In a qualitative approach, this could translate into a “very low” level of risk. In standard-dose chest CT, the estimated LAR is 15–195 for cancer incidence and 14–40 for cancer mortality per 100,000 people that are consistent with a “low” level of risk. Note that these risk estimates depend on multiple factors, viz. patients’ age at exposure, sex and scan settings used for data acquisition, with higher risk for females and younger individuals [33]. Thus, CT operators/technologists need to be aware of various techniques aimed to reduce radiation exposure in chest CT without compromising the diagnostic quality of images [106].

## 7. Projected Number of Future Cancers That Could Be Related to Chest CT Scans Performed during COVID-19 Pandemic Worldwide

The BEIR VII report develops the most up-to-date and comprehensive method to estimate the age- and sex-specific LAR of cancer incidence and mortality per 100,000 persons exposed to a single dose of 100 mSv [33]. The LAR can be calculated for specific cancer sites and for all cancers combined. Although organ-specific doses may be more appropriate for estimating the radiation-related cancer risk, the total effective dose can be used with some modifications [50]. Smith-Bindman et al. developed an adjusted method for estimating the LAR of cancer for chest CT using total effective dose and reported a high agreement level between those from the organ-specific method and total effective dose method (r_c_ = 98%, 95% CI = 96%, 99%) [50]. Therefore, we used this adjusted method to calculate the age- and sex-averaged LAR of cancer incidence and mortality for all cancers combined for the range of total effective doses presented in Table 4 and Table 5.

Globally, from the beginning of the pandemic to date, 616,965,416 confirmed cases of COVID-19, including 6,530,305 deaths, have been reported to the WHO [19]. From Table 4 and Table 5, it can be derived that a typical low- and standard-dose chest CT delivers an effective dose of 0.20–1.8 mSv and 2.20–8.70 mSv to the patients, respectively, depending on the patient’s age at exposure and scan settings used. Using the BEIR VII preferred risk estimation model, in a hypothetical scenario, if we presume that each surviving person infected by COVID-19 was exposed to a single standard-dose chest CT, 472,500–1,868,500 new cases of cancer incidence and 237,300–938,400 cancer deaths could be expected in the near future. Low-dose chest CT could decrease this risk to 42,950–386,600 cases of cancer incidence and 21,500–194,100 cancer deaths. However, in reality, since the number of COVID-19 patients who underwent CT examination was much less than the total number of COVID-19-positive subjects; there will be a proportionate decrease in the estimated cancer incidence and number of deaths, and this will take these estimated figures to a minuscule fraction. Moreover, a few of these patients may not survive long enough to develop and show any carcinogenic effects of radiation in the future.

## 8. Radiation Risk from Chest CT ‘in Perspective’

The radiation risks associated with medical imaging, especially CT scanning [107], have always been exaggerated in the media, which spreads fear and misperception in the population about CT procedures [3]. Therefore, we have attempted to compare the small LAR of cancer incidence and mortality associated with chest CT to other radiation sources, daily life risks and LBR.

The annual average radiation exposure per person from all radiation sources is ~3 mSv worldwide, of which 2.4 mSv (80%) is from natural background radiation, 0.59 mSv (19.7%) is from medical exposures, and 0.01 mSv (0.3%) is from other man-made radiation sources [8,108]. In some areas of Brazil, India, Iran and China, the annual natural background radiation is significantly more than 2.4 mSv [109,110]; nonetheless, no increase in cancer risk has been reported in their inhabitants [110]. In comparison, the lifetime risk of a person dying in a motor vehicle accident is ~1% [111], the risk of a severe allergic reaction due to intravenous contrast media is 0.18% [112] and the sex-averaged LBR for cancer incidence is ~42% and for cancer mortality is 20% [8,33]. The LAR of cancer death from a commercial air flight of 4500 miles is comparable with the risk of cancer death from a low-dose chest CT, whereas driving 2000 miles has a risk of death equivalent to a standard-dose chest CT [61]. 

## 9. Conclusions

Chest CT has been used extensively during the COVID-19 pandemic. Typical low- and standard-dose chest CT delivers an effective dose of 0.20–1.8 mSv and 2.20–8.70 mSv to the patient, which could translate into ‘very low’ and ‘low’ level of radiation-induced cancer risk, respectively. However, for low-dose radiation exposure from medical imaging such as CT, there is no consensus on LAR estimates, and the understanding of radiation biology and radiation-induced cancer risk is still evolving. However, the role of CT scans in patient care cannot be undermined, as the plethora of clinical benefits that the CT provides far outweighs the small hypothetical cancer risk associated with it. Nevertheless, CT should be used judiciously and only when clinically indicated, keeping in mind the ALARA principle, and every attempt should be made to avoid unnecessary and repeat scans.

## Figures and Tables

**Table 1:** A qualitative approach to communicate different levels of cancer incidence associated with radiation exposure compared with the lifetime baseline risk of cancer incidence.

Risk Qualification	LAR of Cancer Incidence per 100,000 People	LBR ^a^ (%)	% LBR + % LAR ^b^
Negligible	<0.2	42	42.00
Minimal	0.2–2	42	42.00
Very low	2–20	42	42.02
Low	20–200	42	42.25
Moderate	200–400	42	42.50

LAR: Lifetime attributable risk; LBR: lifetime baseline risk. ^a^: Sex-averaged lifetime attributable risk of cancer incidence in general population; ^b^: probability of cancer incidence in general population. Adopted with permission from Ref. [8]. 2019, World health organizations.

**Table 2:** A qualitative approach to communicate different levels of cancer mortality associated with radiation exposure compared with the lifetime baseline risk of cancer mortality.

Risk Qualification	LAR of Fatal Cancer per 100,000 People	LBR ^a^ (%)	% LBR + % LAR ^b^
Negligible	<0.1	20	20.00
Minimal	0.1–1	20	20.00
Very low	1–10	20	20.01
Low	10–100	20	20.10
Moderate	100–200	20	20.20

LAR: lifetime attributable risk; LBR: lifetime baseline risk. ^a^: Sex-averaged lifetime attributable risk of fatal cancer in the general population; ^b^: probability of fatal cancer in the general population. Adopted with permission from Ref. [8]. 2019, World health organizations.

**Table 3:** The BEIR VII preferred estimates of the lifetime attributable risk of cancer incidence and mortality from exposure to 100 mSv per 100,000 persons (95% subjective confidence interval).

	All Solid Cancers		Leukemia	
	Males	Females	Males	Females
Excess cancer cases	800 (400–1600)	1300 (690–2500)	100 (30–300)	70 (20–250)
Excess deaths	410 (200–830)	610 (300–1200)	70 (20–220)	50 (10–190)

Adapted with permission from Ref. [33]. 2022, Biologic Effects of Ionizing Radiation (BEIR) VII report.

**Table 4:** The main scan settings, radiation doses and sex-averaged lifetime attributable risk of cancer incidence and mortality from low-dose chest CT for COVID-19 in the literature.

Ref	LAR of Cancer per 100,000 Persons *		ED (mSv)	DLP (mGy.cm)	CTDI_vol_ (mGy)	Pitch	mA/mAs	kVp	Sample Size	Mean Age [Range] (Year)
	Mortality	Incidence								
[84]	5.5	7.5 ^c^	1.80	112	3.50	1	30 ^a^	120	20	64 [≥50]
[85]	2.3	3.7	0.56	40	1.27	1.2	21.5 ^a^	100, 120	192	61.8
[23]	3.6	6.1	0.91	64.7	1.77	1.5	20, 30 ^b^	110, 120	163	65 [21–97]
[86]	3.7	6.7	0.85	61	1.6	1.4	45 ^a^	120	250	50 [16–84]
[86]	2.6	4.7	0.59	42	1.1	1.4	22 ^a^	120	250	50 [16–84]
[87]	1.1	1.9	0.28	20.4	-	1.5	35–50 ^b^	80	250	60 [18–97]
[83]	1	1.5	0.20	14.2	0.39	1.7	10 ^b^	100	380	66.3 [>18]
[88]	2.5	4.3	0.56	40.3	1	1.37	50 ^b^	100	141	37

kVp: kilovoltage peak, mA: miliamper, CTDI_vol_: volume computed tomography dose index, DLP: dose-length product, ED: effective dose, LAR: lifetime attributable risk.* Calculations are made using sex-averaged LAR of cancer incidence and mortality from the BEIR VII report [33]. ^a^. mAs, ^b^. mA, ^c^. LAR estimations are given by the reference.

**Table 5:** The main scan settings, radiation doses and sex-averaged lifetime attributable risk of cancer incidence and mortality from standard-dose chest CT in the literature.

Ref	LAR of Cancer per 100,000 Persons *		ED (mSv)	DLP (mGy.cm)	CTDI_vol_ (mGy)	Pitch	mAs	kVp	Sample Size	Mean Age [Range] (Year)
	Mortality	Incidence								
[84]	20.3	27.1 ^a^	6.6	413	13	1	150	120	20	64 [≥50]
[89]	28.6	50	6.6	415	9.50	-	100	120	180	41.5 [18–74]
[77]	-	195 ^a^	-	-	-	-	-	-	-	15
[8]	-	150 ^a^	-	-	-	-	-	-	-	[≤15]
[25]	19	28.2	5.3 ^b^	329	8	-	-	100–130	782	59
[90]	-	15.18 ^a^	2.2	-	-		-	-	5746	[≤5]
[91]	17.25	29.4	4.3	318	9	1.3	100	120	691	66 [20–≥80]
[92]	31	55.6	7	650	8.8	0.9–1	168–350	120	200	[15–80]
[93]	17.4	21 ^a^	4.4	239	6.8	1.2	132	110, 120	3224	67 [17–105]
[94]	14	16.1 ^a^	3.1	-	-	1.2, 1.4		130	1003	[>12]
[17]	20.7	33.2	5	355	10.5	0.7–1.5	-	80–120	550	47
[50]	34.5	87 ^a^	8.7	-	-			-	120	[≥18]
[95]	40	51.3 ^a^	3.8	-	-	1.42	40	120	765	[≤15]
[88]	20.4	35.4	4.6	330	8	1.37	90–400	120	92	40

kVp: kilovoltage peak, mA: milliampere, CTDI_vol_: volume computed tomography dose index, DLP: dose-length product, ED: effective dose, LAR: lifetime attributable risk. * Calculations are made using sex-averaged LAR of cancer incidence and mortality from the BEIR VII report [33]. ^a^. LAR estimations given by the reference. ^b^. Effective dose derived from DLP using a conversion factor of 0.016 mSv/mGy.cm [84,94].

## Data Availability

The data presented in this review article can be retrieved from the references detailed below.
